# A Case Report of Fascicular Ventricular Tachycardia in a COVID-19 Patient

**DOI:** 10.7759/cureus.31618

**Published:** 2022-11-17

**Authors:** Fakhruddin Almuzghi, Muataz Omar Kashbour, Abdulrahman Almalti

**Affiliations:** 1 Internal Medicine Department, Misrata Medical Center, Misrata, LBY; 2 General Practice, Faculty of Medicine, Misurata University, Misrata, LBY

**Keywords:** ventricular tachycardia, verapamil, case report, covid-19, fascicular ventricular tachycardia

## Abstract

A 29-year-old man with moderate coronavirus disease 2019 (COVID-19) pneumonia presented with a one-day history of palpitations. On examination, he was febrile and tachycardic (pulse rate of 182 beats per minute), with a blood pressure of 120/80 mmHg and oxygen saturation of 96%. Electrocardiography revealed sustained monomorphic wide-complex tachycardia. Carotid sinus massage and adenosine administration were ineffective. Although amiodarone administration slowed the heart rate and relieved his symptoms, sinus rhythm was not restored. We administered intravenous verapamil which terminated his arrhythmia. We diagnosed the patient with fascicular ventricular tachycardia (VT) with a right bundle branch block. He recovered from COVID-19 weeks later. The workup excluded all possible risk factors associated with VT except for COVID-19 infection.

## Introduction

Most cases of ventricular tachycardia (VT) encountered in emergency departments are ischemic in origin [1]. Fascicular VT is an uncommon type of VT that was first discovered by Belhassan in 1979. This benign VT is typically sensitive to verapamil and is referred to as Belhassen or verapamil-sensitive VT [2]. Reportedly, this arrhythmia has a re-entry circuit mechanism in the Purkinje fibers, which contributes to its characteristic electrocardiographic (ECG) pattern of wide-complex tachycardia (WCT) with a right bundle branch block (RBBB) and left axis deviation [3]. Epidemiological data on this subtype of arrhythmia are unavailable; therefore, the incidence of fascicular VT in middle-aged patients remains unknown, which may be attributable to the rarity of this condition or underreporting by physicians. Here, we discuss a rare clinical presentation of a moderate coronavirus disease 2019 (COVID-19) infection accompanied by fascicular VT.

## Case presentation

**Figure 1:**
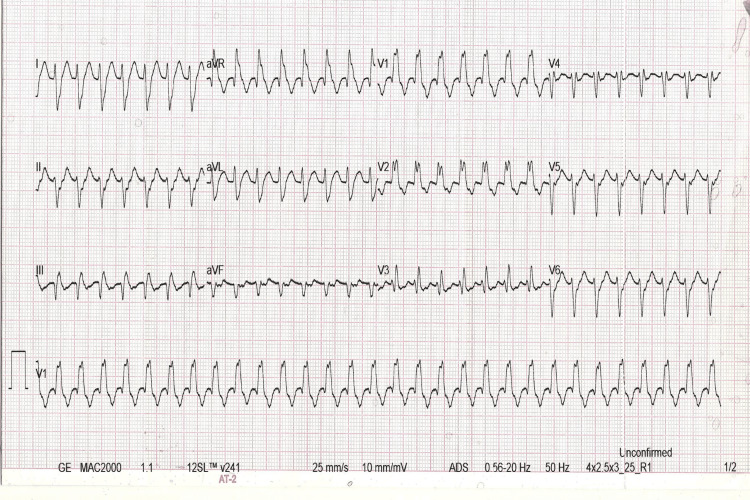
ECG 1: A 12-lead ECG of the patient at presentation. ECG: electrocardiogram

A 29-year-old male physician presented to our emergency department for evaluation of palpitations, pleuritic chest pain and tightness, as well as dyspnea, mild cough, and fever. He developed palpitations one day prior and observed the other symptoms only a few hours prior to presentation. He sought medical attention owing to worsening palpitations and chest tightness. He denied a history of similar symptoms or syncope. He acknowledged recent contact with patients diagnosed with COVID-19 and denied receiving the COVID-19 vaccine. On examination, he was conscious and oriented with a blood pressure of 120/80 mmHg, a regular pulse at 182 beats per min (bpm), oxygen saturation of 96% on room air, and temperature of 38.5°C. Lung auscultation revealed crepitations bilaterally. The remaining physical examination was unremarkable. ECG showed sustained monomorphic WCT, heart rate 182 bpm, QRS complex 180 ms, and no capture of fusion beats (Figure 1). Therefore, our working diagnosis was WCT secondary to clinically diagnosed COVID-19 pneumonia.

**Figure 2:**
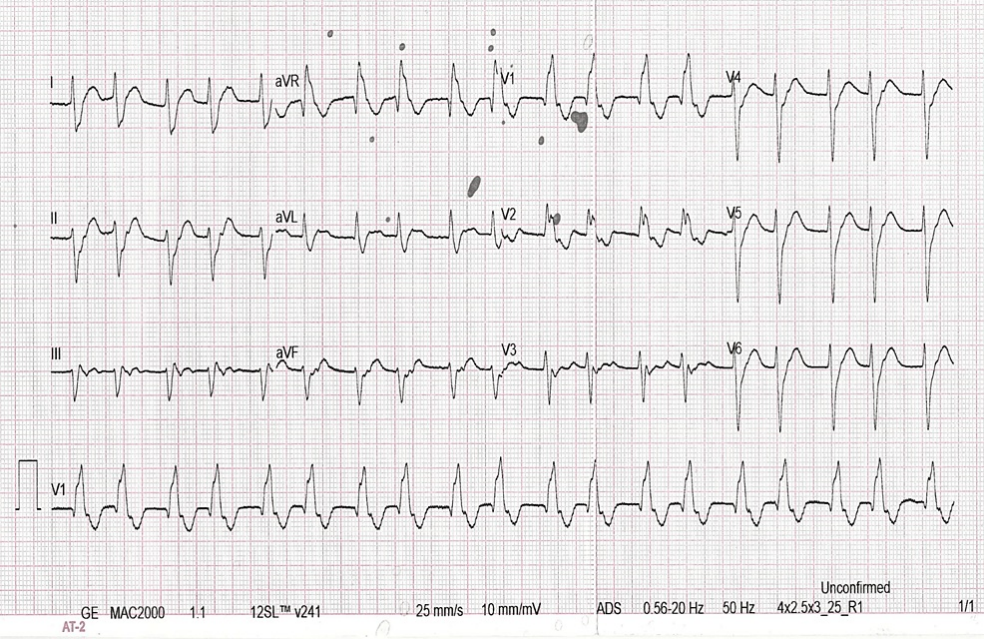
ECG 2: A 12-lead ECG of the patient after the administration of 150 mg of amiodarone intravenously. ECG: electrocardiogram

We performed carotid sinus massage and administered intravenous adenosine (6 mg initially followed by 12 mg), which were ineffective. Subsequently, we administered intravenous amiodarone (150 mg), which led to a decrease in the heart rate from 182 bpm to 120 bpm, with a resolution of palpitations. However, he continued to show a ventricular rhythm (Figure 2). We observed a decrease in the pulse rate compared with the heart rate (61 bpm vs. 121 bpm) (Video 1).

**Video 1:** Telemetry showing the discrepancy between pulse rate (61 bpm) and heart rate (122 bpm) after amiodarone administration. bpm: beats per minute

**Figure 3:**
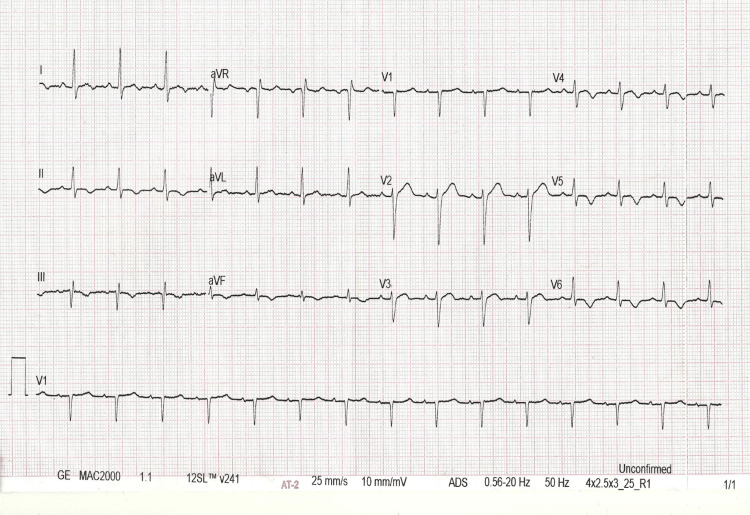
ECG 3: A 12-lead ECG of the patient after administration of 10 mg of verapamil intravenously. ECG: electrocardiogram

The dyssynchrony between the heart and pulse rate was not evident before amiodarone treatment. The patient refused electrical cardioversion. He had a cardiology consultation and was diagnosed with fascicular VT based on the findings of WCT, RBBB, left axis deviation, and failure to restore sinus rhythm despite amiodarone therapy. Following the cardiology consultation, we administered intravenous verapamil (10 mg), which successfully terminated the arrhythmia and restored normal sinus rhythm in less than one minute (Figure 3, Video 2).

**Video 2:** Telemetry showing normal sinus rhythm after verapamil administration.

**Figure 4:**
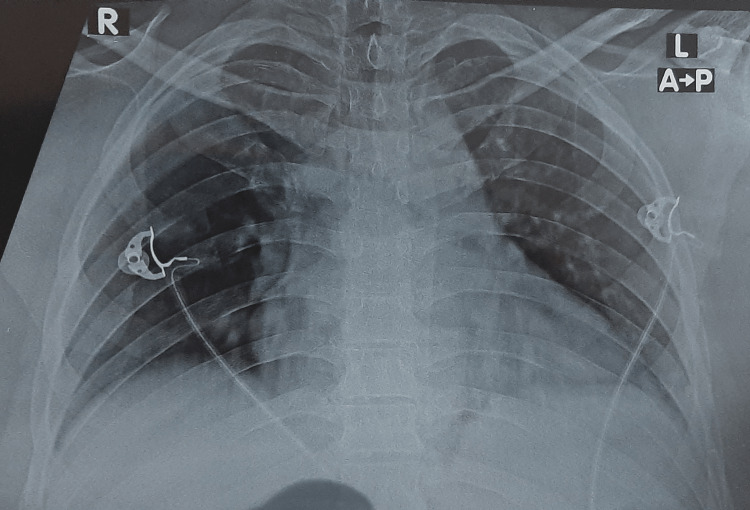
Anteroposterior chest X-ray showing bilateral pulmonary opacification.

Blood test results showed leukocytosis with lymphopenia, an elevated serum C-reactive protein (CRP) level, and slightly elevated troponin levels (Table 1). Chest radiography showed bilateral pulmonary opacification (Figure 4).

**Table 1:** Laboratory results. CBC: complete blood count; WBC: white blood cell; MCV: mean corpuscular volume; MCH: mean corpuscular hemoglobin; MCHC: mean corpuscular hemoglobin concentration; RDW-CV: red cell distribution width; RDW-SD: red cell distribution width standard deviation; CRP: C-reactive protein; ALT: alanine aminotransferase; AST: aspartate aminotransferase; ALP: alkaline phosphatase; CK-MB: creatine kinase-MB; TSH: thyroid-stimulating hormone

CBC		Biochemistry	
WBC	16.7 × 10^9^/L	Glucose	114 mg/dL
Lymphocytes (number)	1.9 × 10^9^/L	Urea	25 mg/dL
Mid (number)	0.9 × 10^9^/L	Creatinine	0.83 mg/dL
Granulocytes	13.9 × 10^9^/L	CRP	35.3 mg/dL
Lymphocytes (%)	11.4%	Procalcitonin	0.039 ng/ml
Mid (%)	5.1%	ALT	19.1 U/L
Hemoglobulin	12.9 g/dL	AST	14 U/L
RBC	4.22 × 10^12^/L	Albumin	4.55 g/dL
Hematocrit	36 %	ALP	67.3 U/L
MCV	85.5 fL	Total bilirubin	0.86 mg/dL
MCH	30.5 pg	Na	143 mmol/L
MCHC	35.8 g/dL	K	4.8 mmol/L
RDW-CV	12.7%	Cl	106 mmol/L
RDW-SD	43.6	Ca	10.3 mg/dL
Platelets	210 × 10^9^/L	Mg	1.8 mg/dL
		Troponin	0.220 ng/mL
		CK-MB	1.43 ng/mL
		D-dimer	0.27 ug/ml
		TSH	2.99 uIU/ml

At his request, the patient was discharged after a few hours of observation. We prescribed long-acting verapamil for one week and referred the patient to a cardiologist. Transthoracic echocardiography was normal. Cough and fever were worse during the one-week follow-up and resolved completely at the two-week follow-up. COVID-19 serology tests showed positive results a few weeks later.

## Discussion

Although many cardiologists successfully identify fascicular VT, accurate diagnosis of this VT subtype may be challenging for emergency department physicians [4]. Failure to restore sinus rhythm despite amiodarone administration may be a useful diagnostic clue; amiodarone is reportedly effective for this VT subtype [5]. We followed the Advanced Cardiac Life Support algorithm for WCT for managing our patient. Unresponsiveness to Valsalva maneuvers and adenosine therapy favored a diagnosis of VT, and supraventricular tachycardia (SVT) was less likely in this case. Considering the potential adverse outcomes associated with verapamil in VT management, we administered intravenous amiodarone [6,7]. Although amiodarone significantly decreased tachycardia and resulted in symptomatic relief, sinus rhythm was not restored in this patient; therefore, we concluded that this was not a typical case of VT.

Previous studies have reported the effectiveness of verapamil in the management of fascicular VT [2,8]. Normal sinus rhythm was restored in our patient only after intravenous verapamil administration. It is important to remember that WCT that responds immediately to verapamil administration may be misdiagnosed as SVT instead of fascicular VT by emergency department clinicians. Therefore, it is necessary to determine the true incidence of this variant, which is invariably missed or misdiagnosed. Accurate diagnosis of fascicular VT is essential because the ideal drug administered for this ventricular arrhythmia subtype (verapamil) may precipitate cardiac arrest in stable patients with other types of VT [6,7]. Moreover, fascicular VT shows a good prognosis; therefore, patient education can enable rapid identification and prompt initiation of treatment in cases of recurrence [9].

Studies have reported the onset of VT in patients with critical COVID-19 [10]. This arrhythmia is attributable to systemic illness and not exclusively to COVID-19 infection [11]. Interestingly, our patient did not show hypoxia or evidence of a systemic inflammatory response syndrome at presentation. A similar presentation has been reported in the literature [12,13]. A woman developed right ventricular outflow tract VT alternating with sinus rhythm that was successfully treated using beta-blocker therapy [12]. The patient had intermittent episodes of palpitations in contrast to persistent palpitations for approximately one day, as observed in our patient. Both patients had mild-to-moderate COVID-19 infection; however, comorbidities that may have triggered VT were not observed in either patient. Notably, both patients presented to the hospital primarily for evaluation of palpitations (fascicular VT) and not with the usual symptoms of COVID-19.

A limitation of our study is that we clinically diagnosed our patient with COVID-19 infection based on a history of contact with patients with COVID-19 and findings of fever, dry cough, leukocytosis, lymphopenia, elevated serum CRP, imaging findings, and elevated severe acute respiratory syndrome coronavirus 2 immunoglobulins and not based on reverse transcription-polymerase chain reaction (RT-PCR) assay results.

## Conclusions

Fascicular VT is a rare type of VT. In this study, we report a clinical presentation of COVID-19 infection with fascicular VT. Vagal maneuvers and intravenous adenosine failed to terminate arrhythmias. Although intravenous amiodarone slowed the heart rate and relieved symptoms, it did not restore sinus rhythm. We administered 10 mg of intravenous verapamil, which restored sinus rhythm. The diagnosis of COVID-19 infection was made clinically and not by RT-PCR. The patient recovered from COVID-19 weeks later, and the COVID-19 immunoglobulin test was positive. Except for the COVID-19 infection, this healthy middle-aged patient had no risk factors for arrhythmia. Further analytical research is warranted to definitively validate the association between mild-to-moderate COVID-19 and fascicular VT.
